# Comparative proteomic analysis of compartmentalised Ras signalling

**DOI:** 10.1038/srep17307

**Published:** 2015-12-01

**Authors:** Maria Hernandez-Valladares, Ian A. Prior

**Affiliations:** 1 Physiological Laboratory, Institute of Translational Medicine, University of Liverpool, Crown Street, Liverpool L69 3BX, United Kingdom

## Abstract

Ras proteins are membrane bound signalling hubs that operate from both the cell surface and endomembrane compartments. However, the extent to which intracellular pools of Ras can contribute to cell signalling is debated. To address this, we have performed a global screen of compartmentalised Ras signalling. We find that whilst ER/Golgi- and endosomal-Ras only generate weak outputs, Ras localised to the mitochondria or Golgi significantly and distinctly influence both the abundance and phosphorylation of a wide range of proteins analysed. Our data reveal that ~80% of phosphosites exhibiting large (≥1.5-fold) changes compared to control can be modulated by organellar Ras signalling. The majority of compartmentalised Ras-specific responses are predicted to influence gene expression, RNA splicing and cell proliferation. Our analysis reinforces the concept that compartmentalisation influences Ras signalling and provides detailed insight into the widespread modulation of responses downstream of endomembranous Ras signalling.

Ras proteins are low molecular mass GTPases that operate as molecular switches near the top of signalling cascades that regulate several cellular functions including proliferation, differentiation, migration and apoptosis^1^. They are encoded by 3 ubiquitously expressed genes: HRAS, KRAS (with spliced isoforms KRAS4A and KRAS4B) and NRAS that are frequently mutated in human cancers^2^. Alignment of the amino acid sequences of the Ras isoforms reveals that all of the regions required for GDP/GTP binding and effector interactions are essentially identical. The only region of significant difference is the C-terminal hypervariable region (HVR) that is subjected to sequential post-translational modifications defining isoform-specific membrane binding and trafficking^3^. The *S*-farnesylation on the cysteine residue at the C-terminal CAAX motif is responsible for a weak binding to the endoplasmic reticulum (ER) membranes^4^. However other motifs within the HVR are essential for the nanolocalisation of each isoform. For KRAS4B, a hexa-lysine polybasic domain directs targetting to the plasma membrane while the presence of basic patches and palmitoylation/de-palmitoylation events on adjacent cysteine residues facilitate the correct trafficking of KRAS4A, HRAS and NRAS between organelle and plasma membranes^4^,^5^,^6^,^7^,^8^.

Despite a high degree of sequence homology and ubiquitous expression profiles, Ras isoforms are not biologically redundant and generate different signal outputs. These include distinct contributions to embryonic development, cancer development and differential coupling to canonical effector pathways^9^,^10^,^11^,^12^. These signalling differences could be at least in part due to a differential trafficking and localization of each isoform within the cell surface and endomembranes allowing differential access to pools of activators and effectors^13^.

Good examples of compartmentalised Ras signalling have already been provided. For example, endogenous Golgi-dependent Ras signalling regulates thymocyte selection^14^. Ras signalling restricted to endomembranes has been demonstrated to regulate a variety of classical Ras effectors and phenotypic outputs in mammalian and yeast cells^15^,^16^,^17^,^18^,^19^,^20^. It is also clear that interfering with normal Ras trafficking and localisation results in impaired signalling^21^,^22^. Whilst there is a general consensus that spatial context can influence Ras signalling, there are still poor understanding of the pathways that will be directly or indirectly modulated by compartmentalised Ras. In part, this is due to the challenges associated with directly measuring Ras signalling associated with specific locations.

To measure compartmentalised Ras signalling, we have used constitutively active (G12V) Ras variants engineered to localise to each of the cellular locations where has been demonstrated to functionally operate^19^. Using SILAC-based quantitative phosphoproteomics to measure the network response, we have quantified distinct outputs associated with Ras signalling from each location. This study represents the first attempt to define the extent to which compartmentalised Ras signalling modulates nodes within the normal Ras signalling network.

## Results

### Intracellular localisation of Ras chimeras

**Figure 1:**
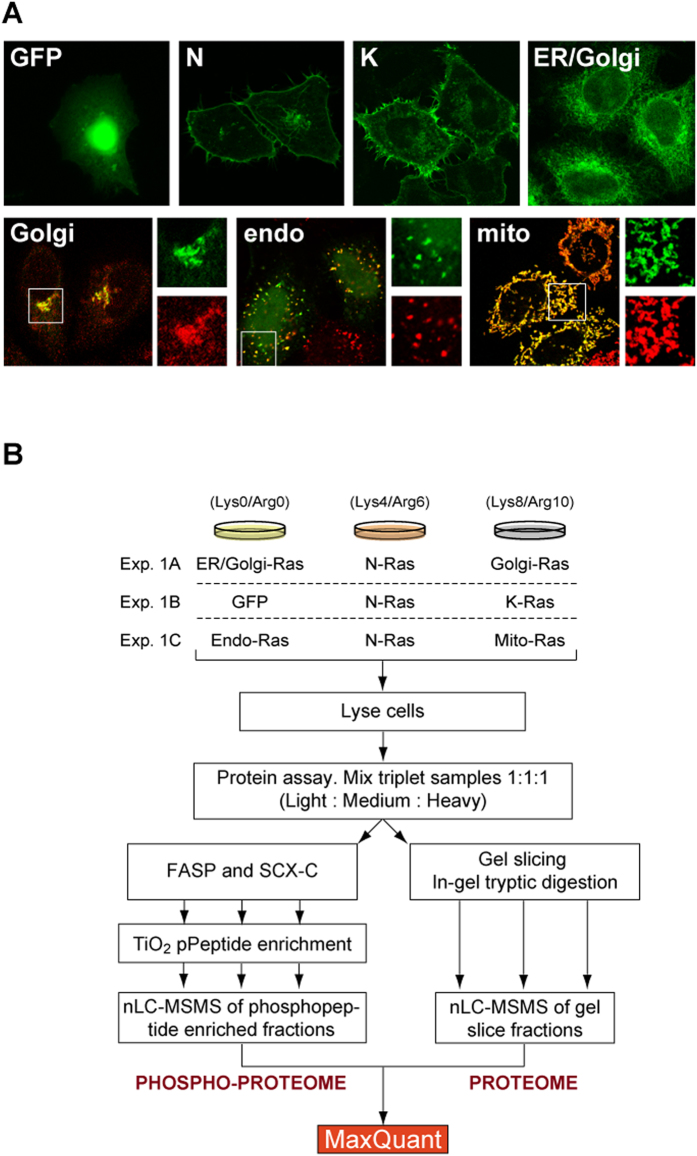
Organellar Ras and experimental scheme. (**A**) Fluorescence microscopy images showing subcellular locations of transiently expressed Ras (G12V) mutant constructs in HeLa S3 cells. Anti-PDI1, anti-Grasp55, anti-EEA1 and anti-TOM20 antibodies (red) were used to confirm ER, Golgi, endosomal and mitochondrial location, respectively. (**B**) Workflow for the peptide and phosphopeptide preparations used in this study. The triplex organisation for a single biological replicate is shown.

To investigate location-specific Ras signalling, we utilised a series of GFP-tagged constitutively active (G12V) Ras chimeras where the C-terminal Ras membrane targeting and localisation motifs has been replaced by organelle-specific targetting motifs^19^. As positive controls for normal Ras localisation and function we used NRAS and KRAS (Fig. 1A). KRAS displays a predominant plasma membrane distribution with a small fraction on the ER and cytosol. In contrast, NRAS exhibits a significant endomembrane and Golgi pool in addition to cell surface localisation. ER/Golgi, Golgi and mitochondrial Ras are highly restricted to their target organelles based on morphology and/or co-localisation with organelle markers. Endosomal Ras is the least specifically targeted; however, clear co-localisation with the early endosomal marker EEA1 can be seen whilst the nucleus localisation is likely to be unproductive and therefore silent in our study (Fig. 1A).

Stable isotope labelling with amino acids in cell culture (SILAC) allows cell populations representing individual experimental points to be selectively labelled with isotopes of arginine or lysine for quantitative proteomic analysis. Cell lysates were mixed 1:1:1 and run directly on SDS-PAGE gels for proteome analysis or subjected to strong cation exchange-based fractionation and TiO_2_-based phosphopeptide enrichment procedures (Fig. 1B). Due to the number of conditions, HeLa cells transfected with GFP-NRAS (G12V) were used as a common point in each triplex experiment to allow pairwise comparisons between all conditions. Across three biological replicates, similar transfection efficiency and expression of each Ras variant was observed (Supplementary Figure 1). The exception to this was GFP-NRAS (G12V) that consistently exhibited >2-fold more expression than the other variants.

### Proteome and phosphoproteome profiles of organellar Ras

In total, 17,205 responses were measured across the proteome and phosphoproteome datasets averaged from three biological replicates (Supplementary Table 1). These related to 1,353 unique proteins in the proteome dataset and 2,152 unique phosphosites quantified from 1,252 proteins in the TiO_2_ enrichments. Ratios for all 6 Ras proteins versus the GFP control were present for 891 proteome dataset proteins and 1356 class 1 phosphosites (localisation probability ≥0.75); these data points were used for all subsequent analysis.

**Figure 2:**
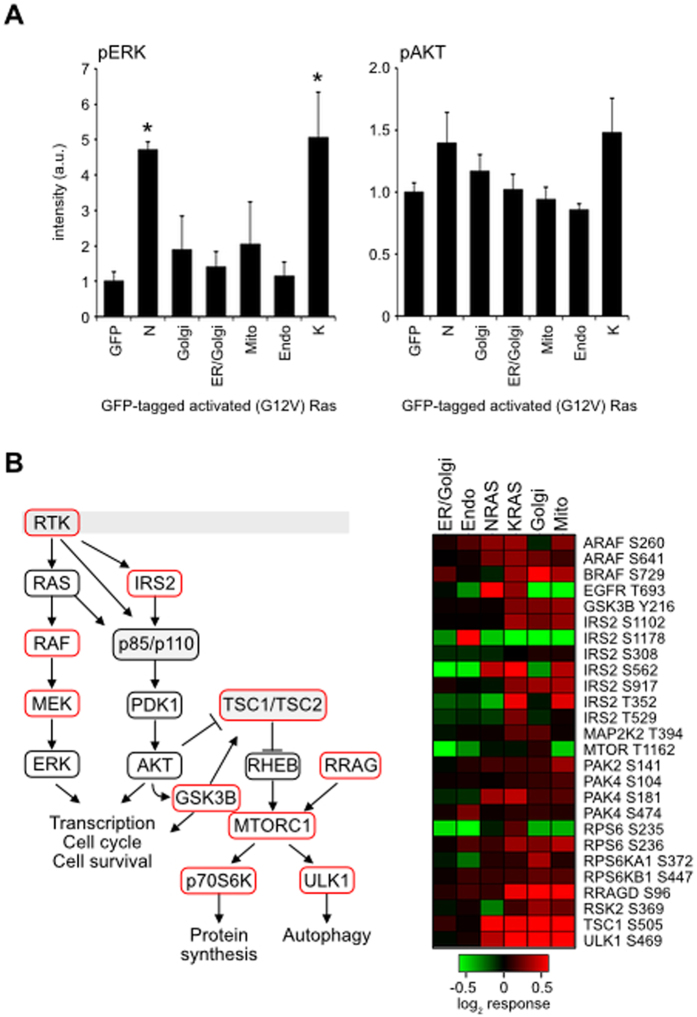
Responses within the local Ras signalling network. (**A**) Ras effector activation downstream of compartmentalised Ras. Mean values +/− SEM of western blot quantitation of phospho-ERK and phospho-AKT normalised to actin are presented. n = 4, *p < 0.05 paired two-tailed T-test. (**B**) Nodes identified in the phosphoproteome dataset are highlighted in red and exhibit highly distinctive compartment-specific responses.

The network response to Ras activation is likely to be significantly influenced by the signal flow through the Raf-MAP kinase and PtdIns-3-kinase-AKT effector pathways. To provide initial insight into the activation of these canonical effector pathways we used western blotting for phospho-ERK and phospho-AKT and found that whilst NRAS and KRAS elicit potent MAP kinase activation, the organellar Ras locations exhibit weak trends for increased outputs (Fig. 2A). Similar trends are seen for phosphor-AKT responses. Despite the apparently weak outputs associated with the organellar Ras variants examination of collation of the responses of the local Ras network nodes within the phospho-proteomic data revealed organelle-specific responses throughout the network (Fig. 2B). Notably, ER/Golgi-Ras and endo-Ras appeared to be highly distinct from the other Ras variants.

**Figure 3:**
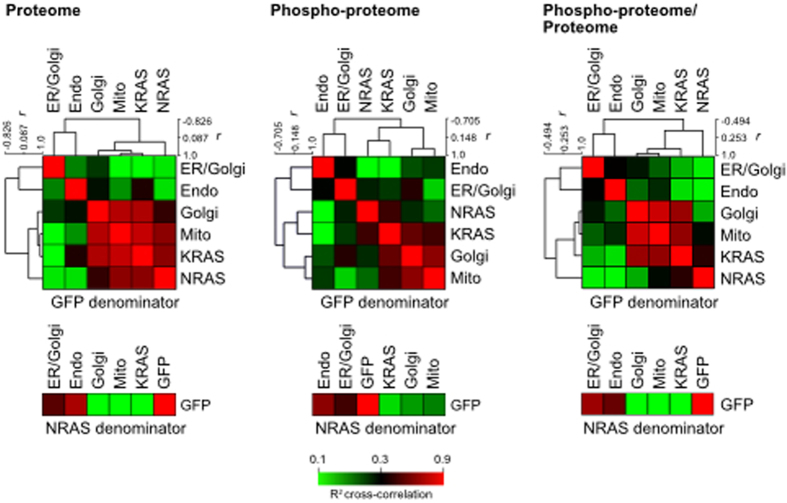
Compartment-specific Ras network responses. Proteome and phosphoproteome cross-correlations among six Ras (G12V) distinct organellar locations using either GFP or NRAS (G12V) in the denominator of log2-transformed intensity ratios. High correlation values (near 1.0) were red-coloured and clustered together. The influence of changes in protein abundance in the proteome on the their cognate phosphosite responses was removed in the final cross-correlation matrix (Phospho-proteome/Proteome) to reveal high correlation between KRAS, Golgi-Ras and mito-Ras phosphosite responses. Analysis was performed on ratios from MaxQuant analysis of n = 3 biological repeats.

To provide a comprehensive overview of organelle response variability we performed cross-correlation and hierarchical clustering with protein and phosphosite ratios using GFP values in the denominator (Fig. 3). Two distinct groups were observed: one that involves Golgi-, Mito-, K- and N- (G12V) Ras and another that involves ER/Golgi- and Endo- (G12V) Ras. Removing the influence of the protein abundance changes within the proteome strengthened the correlation between KRAS, Golgi-Ras and mito-Ras in the phosphosite dataset. When SILAC ratios were calculated using NRAS as the denominator, both Endo and ER-Golgi Ras outputs correlate with GFP (Fig. 3). The similarity to the negative control indicates that these two locations do not support robust Ras signalling. In contrast, the Golgi and mitochondria sustain distinctive responses that do not correlate with the GFP negative control. In this respect they resemble the positive controls of NRAS and especially KRAS, suggesting that these two organelles are capable of supporting significant Ras signalling outputs. These data reveal that each location generates a distinct and network response; that KRAS, NRAS, Golgi-Ras and mito-Ras outputs are robust and broadly similar and that endo-Ras and ER/Golgi-Ras are weak activators of the Ras network.

**Figure 4:**
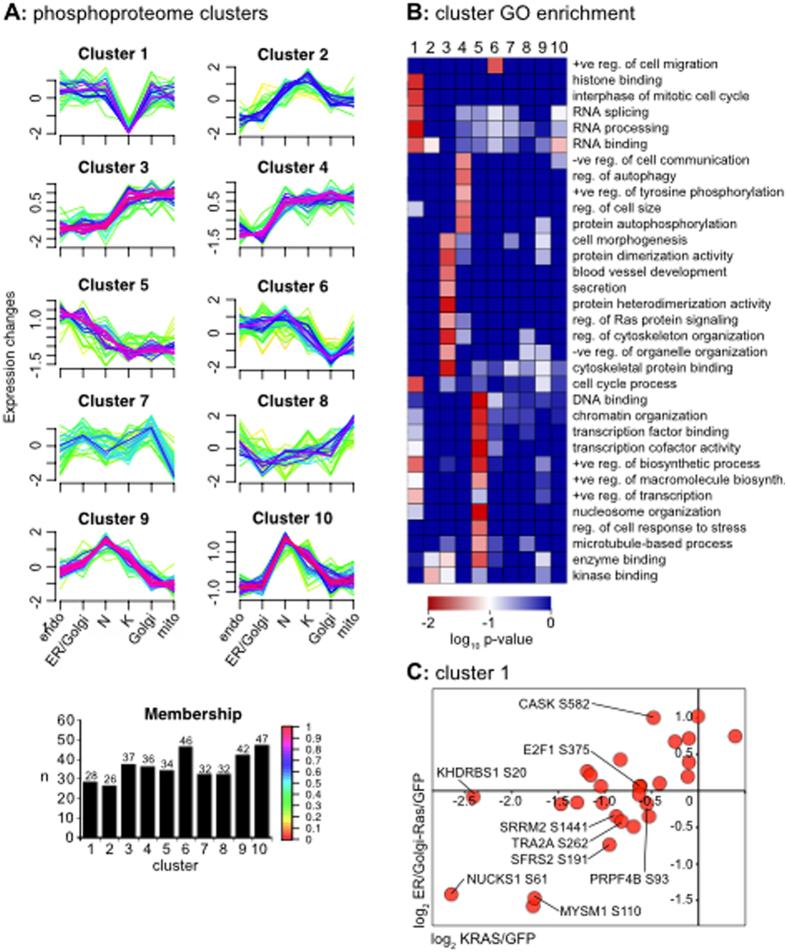
Phosphosites displaying organelle-specific responses. (**A**) Ratios exhibiting changes in abundance were subjected to unsupervised clustering with the Fuzzy c means algorithm using GProX. Clusters corresponding to ten different response patterns were identified. The number (n) in each cluster is indicated. (**B**) GO analysis indicates that proteins associated with RNA processing, gene expression, the cytoskeleton and cell proliferation are significantly enriched. (**C**) Members of cluster 1, enriched for RNA processing proteins are highlighted in a representative KRAS versus ER/Golgi-Ras scatter graph.

In order to identify the responses associated with each location, we subjected the datasets to unsupervised fuzzy c-means clustering analysis using GProX^23^. This identifies genes that exhibit matching responses across the phosphoproteome (Fig. 4) or proteome datasets (Supplementary Figure 2 and Supplementary Table 1). Intriguingly, only KRAS exhibits a uniquely coupled subset of responses (Fig. 4A, cluster 1). Gene ontology (GO) analysis reveals a significant enrichment for genes associated with DNA modification, transcription and RNA splicing within the KRAS subset (Fig. 4B). A representative comparison with ER/Golgi-Ras illustrates the reduced relative phosphorylation in cells harbouring KRAS G12V (Fig. 4C). Amongst the KRAS-specific responders is the transcription factor E2F1 that plays an important role in regulating the expression of genes required for DNA replication and cell cycle progression^24^. The most pronounced KRAS-specific response is seen with Ser20 of the transcriptional repressor and p120Ras GAP associated protein KHDRBS1 (SAM68)^25^. Although this site has no described regulatory function, it is responsive to EGF stimulation^26^.

We were specifically interested in organelle-specific Ras stimulated responses. Our earlier data indicated that ER/Golgi and endo-Ras are largely indistinguishable from the GFP control. In contrast, mito-Ras and Golgi-Ras represent robust responders that typically show an equivalent direction of response that is often similar to KRAS. This divergent pattern of responses can be most clearly seen in clusters 2–6 of the GProX analysis (Fig. 4A). Analysis of the phosphoproteome dataset revealed 179 sites associated with clusters 2–6. It has already been seen previously that NRAS phosphosite responses correlate relatively poorly with KRAS, mito-Ras and Golgi-Ras (Fig. 3). This differentiation can be seen in Clusters 3 and 5 that also contribute to the majority of the Gene Ontology terms associated with the dataset (Fig. 4B). Decreased KRAS, Golgi-Ras and mito-Ras network responses are associated with DNA replication, organisation and transcription and the converse associated with cytoskeletal proteins. Whilst all of the responses associated with each cluster can be seen in Supplementary Table 1, clusters 3 and 5 have also been depicted in Supplementary Figure 3.

In summary, the GProX analysis revealed there is no subset of responses uniquely associated with each intracellular location, instead Golgi-Ras, mito-Ras, KRAS and to a lesser extent NRAS responses typically trended together. The majority of nodes responsive to these Ras variants are involved in regulating DNA organisation and replication, transcription cell organisation and the cytoskeleton.

**Figure 5:**
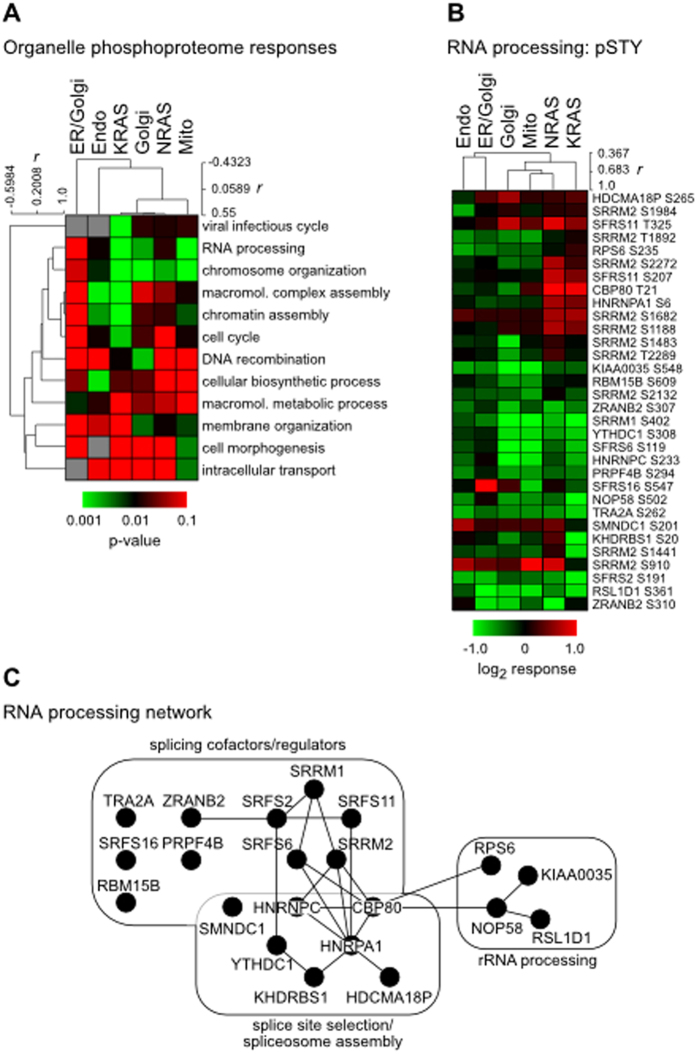
Compartmentalised Ras responsive phosphosites include a network of RNA processing proteins. (**A**) Phosphosites exhibiting ≥1.5-fold responsiveness versus the GFP control were shortlisted and GO analysis indicated that proteins associated with DNA and RNA processing and significantly enriched. (**B**) All of the phosphosites associated with RNA processing proteins are shown; hierarchical clustering indicates similar responses from Golgi, Mito, KRAS and NRAS. Kinase responses from the proteome and phosphoproteome data clustered by cell function ontology. (**C**) STRING analysis of the RNA processing subset reveals an interactome associated with spliceosome function.

An alternative method of collating organelle-specific Ras responses was based on identifying those that substantially differed from the GFP control using an arbitrary 1.5-fold change cut-off. In total we identified 266 responsive phosphopeptides with 215 distinct sites (~80%) being ≥1.5-fold responsive versus the GFP control in at least one of the organellar Ras variants (Supplementary Table 2). GO analysis of the phosphosites exhibiting ≥1.5-fold changes vs GFP indicated that DNA and RNA processing and organisation were major phenotypic targets (Fig. 5A). An illustrative example is depicted using the RNA processing subset of responsive phosphosites (Fig. 5B). The majority of the proteins identified in this subset represent a functional interactome involved in pre-mRNA splice site identification, spliceosome assembly and regulation of RNA splicing (Fig. 5C).

To provide more context for understanding potential information flow within the network, we curated the proteome and phosphosite responses of all of the kinases that together are the master-regulators of the phosphoproteome (Supplementary Figure 4A and B). Whilst none of these phosphosites are located in the kinase active sites, some of them are indicative of signalling status of the kinase. The most prominent example of this and of differentiation between Ras isoforms and organellar Ras can be seen with EGFR T693. Both KRAS and NRAS exhibit increased phosphorylation versus the GFP control whereas the converse is true for all of the organellar-Ras variants (Supplementary Figure 4B). This site is a target for negative feedback regulation of EGFR^27^,^28^, and these data suggest that intracellular Ras variants will be constrained in their ability to down-regulate EGFR. Taken together, our data reveals overlapping but distinctive compartmentalisation-dependent modulation of nodes involved in transcription, translation and cell cycle pathways that are known to be associated with normal Ras signalling.

## Discussion

Whilst the cell surface is the prime location for Ras signalling, endomembrane compartments such as the Golgi, endosome and mitochondria have increasingly been seen as sites of biologically relevant Ras activity^13^,^29^,^30^. Despite this, there is still relatively little insight into the pathways regulated by Ras signalling from these intracellular locations or the extent to which these outputs overlap. A specific challenge in studying this phenomenon is that it is difficult to isolate compartment-specific Ras signalling from the bulk Ras output within the cell. A significant amount of our knowledge of compartmentalised Ras signalling has been derived from studies that tethering activated Ras proteins, Ras effectors or Ras modulators to intracellular locations^17^,^18^,^19^,^31^,^32^. Our own previous work using stably expressed compartment-restricted Ras variants in NIH3T3 cells revealed that all locations were competent for supporting canonical Ras signalling^19^. Whilst over-expression may not faithfully recapitulate relative endogenous Ras densities, this approach reveals the potential range of Ras responses emanating from a specific compartment.

We have used this well-established strategy combined with large-scale quantitative proteomics to generate the first unbiased global analysis of signalling pathways downstream of compartmentalised Ras proteins. We chose to use a transient transfection approach, rather than a stable cell line strategy used previously, to reduce the influence of adaptation to long-term expression. A potential challenge with this approach is that the expression cannot be precisely controlled however we saw consistent expression patterns across biological replicates (Supplementary Figure 1). Our approach has resulted in unprecedented depth of network coverage. Approximately two thirds of the 3500 members of the phosphosite and proteome datasets contained ratios for all six experimental conditions versus a GFP control. Due to the relatively limited effect on proteome responses (<7% of total exhibited ≥1.5-fold changes), we concentrated most data analysis on the phosphosite dataset. Amongst the subset of 266 strongly responsive (≥1.5-fold) phosphosites where we had all 6 ratios, ~80% were observed in at least one of the activated organellar-Ras experimental conditions.

The majority of the nodes within the immediate Ras signalling network displayed differential phosphoproteome responses (Fig. 2). Consistent with the wider dataset, each organellar Ras protein exhibits a distinct response profile. Whilst the majority of the phosphosites measured have no described function, many of the hits within the Ras-mTOR signalling network exhibit increased phosphorylation in response to EGF stimulation and/or dynamic regulation during the cell cycle^33^,^34^. Although we cannot predict likely phenotypic outcomes from these network state differences, these data clearly indicate that organellar Ras outputs impinge on this important signalling nexus.

We consistently observed Golgi-Ras and mito-Ras responses co-clustering with KRAS and to a lesser extent NRAS outputs. In contrast, ER/Golgi-Ras and endo-Ras presented relatively few organelle-specific responses and the cross-correlation analysis indicated that these locations were largely equivalent to the GFP negative control (Fig. 3). This reveals that not all locations can facilitate robust Ras signalling. However, importantly the pattern is not equivalent to the pERK and pAKT profiles associated with the Ras variants (Fig. 2A). This means that the weak network response seen is more likely to be a consequence of subtle cumulative differences in activation across all Ras effectors rather than spatially-regulated uncoupling from effector pathways.

The responses that we observe do not appear to be influenced by the experimental design, ie. co-responsive conditions such as Golgi-Ras, mito-Ras and KRAS were not part of the same triplet. Similarly, they are not a result of expression differences generated by transient transfection approach, ie. there is no correlation between the isoforms showing higher or lower expression (Supplementary Figure 1) and the clustering of responses that we observe.

The inclusion of G12V mutated KRAS and NRAS in the analysis provided an opportunity to compare organellar Ras outputs with the two isoforms most frequently mutated in cancer^2^. KRAS represents a predominantly cell surface associated Ras isoform, whereas NRAS also displays a significant Golgi pool (Fig. 1). It was notable that NRAS displayed relatively low correlation with the other Ras variants (Fig. 3, Supplementary Figures 4A and C). Similarly, KRAS exhibited a unique response with respect to the other Ras variants, largely comprising RNA processing proteins (Fig. 4A, cluster 1). Given that there are no locations where NRAS or KRAS have been observed that are not included in this study, the reason for their unique signatures cannot be due to an untested subcellular pool of NRAS or KRAS. Importantly, the similarities between KRAS and Golgi-Ras argue against pathways uniquely associated with specific locations since both of these Ras proteins exhibit mutually exclusive subcellular distributions^3^. Whilst it is easy to focus only on the significant stimulation of pERK observed with KRAS and NRAS (Fig. 2), our data highlights the how subtle differences in low-grade effector activation generate diverse network responses from each of the Ras variants. Further support for this idea comes from studies where Ras isoforms were shown to differentially activate effector pathways, possibly because they adopt different orientations with respect to the cytosol that influences effector engagement^11^,^12^,^35^.

Examples of KRAS associated responders included E2F1 where the increased phosphorylation of Ser375 that was observed downstream of the compartmentalised Ras proteins versus KRAS is known to increase the transcriptional and cell cycle promoting activity of E2F1^36^. A further example of differential Ras isoform versus organellar Ras responses can be seen with T693 of EGFR (Fig. 2). Although identified as T693 in the MaxQuant analysis, in the post-translationally processed protein it is T669. This residue sits within the juxtamembrane region of EGFR that regulates receptor activation by stabilising the asymmetric dimer of tyrosine kinase domains^27^,^28^. T669 is phosphorylated by ERK and phosphorylation of this site negatively regulates EGFR kinase activity^37^. We observed increased phosphorylation of T699 in the KRAS and NRAS conditions versus the organellar Ras variants (Fig. 2). The simplest explanation for this is that both KRAS and NRAS were also the most potent activators of ERK (Fig. 2). It will be interesting to discover whether the apparently impaired ability of endomembrane Ras to negatively regulate EGFR activity reflects a lack of physical proximity within the pathway or is the result of other accessory and regulatory proteins such as scaffolds and phosphatases being differentially engaged.

GProX analysis (Fig. 4, Supplementary Table 1) and the ≥1.5-fold responsive phosphosite datasets (Fig. 5, Supplementary Table 2) highlighted how there were similar trends between some of the organellar Ras variants but at the level of individual nodes the responses are highly distinctive. GO analysis revealed significant association of DNA organising and cytoskeletal proteins with KRAS, mito-Ras and Golgi-Ras signalling (Fig. 4B, clusters 3 and 5). It will be interesting to see if these proteins differentially affect the cell cycle, gene expression, cell morphology or migration versus NRAS or the other endomembranous Ras proteins.

The variability of responses to each of the organellar Ras proteins is exemplified by SRRM2. SRRM2 is a core member of the spliceosome B* complex involved in the first catalytic step of splicing by the spliceosome^38^. 10 out of 77 SRRM2 phosphosites identified in our dataset exhibited ≥1.5-fold responsiveness. Whilst the majority of the sites showed higher phosphorylation in KRAS and NRAS than the organelle Ras conditions, exceptions to this were evident (S910, S1441 and S2132; Fig. 5B). Furthermore, in all cases the relative magnitudes of the organellar Ras responses varied with respect to each other and the two Ras isoforms.

SRRM2 is a member of the SR protein family, along with SRFS2, SRFS6, SRFS11, SRFS16, ZRANB2, TRA2A and SRRM1 present in our responsive subset (Fig. 5C), that are key regulators of gene expression^39^. This family contain RS domains rich in arginine and serine residues that promotes interactions with proteins and pre-mRNA^40^. RS domains are extensively phosphorylated to control their activity and a dynamic cycle of phosphorylation is needed to promote spliceosome assembly and dephosphorylation to promote spliceosome activity^41^. Therefore, whilst none of the many SR protein phosphosites in our study have been directly investigated for their biological functions, it is tempting to speculate that Ras isoforms and organellar Ras are differentially capable of contributing to spliceosome regulation.

In summary, our approach reveals the potential of an organelle to support Ras signalling. We find that whilst not all locations are effective facilitators of Ras signalling, ~80% of Ras-responsive phosphosites can be modulated by organellar Ras signalling. Furthermore, despite the distinctive responses associated with each Ras protein, there was a clear convergence between Golgi-Ras, mito-Ras and KRAS on the same phenotypic outputs. This demonstrates that Golgi-Ras and mito-Ras are capable of modulating the phenotypes associated with isoform-specific Ras signalling, putatively indicating a fine-tuning role for subcellular Ras. These data reinforce the notion that Ras signalling is highly context dependent and capable of utilising multiple locations within the cell for productive engagement with common effector pathways.

## Materials and Methods

### Materials

HeLa S3 cells were a gift of Francis Barr (University of Oxford, U.K.). DMEM deficient in arginine and lysine, and 10% dialyzed FBS were purchased from Dundee Cell Products (Dundee, U.K). SILAC isotopes, L-lysine-^2^H_4_ (Lys4), L-arginine-U^13^C_6_ (Arg6), L-lysine-U-^13^C_6_-^15^N_2_ (Lys8) and L-arginine-U-^13^C_6_-^15^N_4_ (Arg10) were from Sigma (St. Louis, Missouri). Mammalian protease inhibitors were from Sigma and PhosSTOP phosphatase inhibitor cocktail tablets were from Roche. S-Resource 1 ml column to perform strong cation exchange (SCX) chromatography was from GE Healthcare Life Sciences (Buckinghamshire, U.K). TiO_2_ beads for phosphopeptide enrichment were from GL Sciences (Tokyo, Japan). Mouse anti-Tom20 antibody from BD Biosciences, mouse anti-PDI antibody from Enzo, rabbit anti-EEA1^42^ and sheep anti-Grasp55^43^ were used in colocalization studies. Targeted Ras constructs have been described previously^19^.

### Cell culture and transfection

To generate light (Lys0/Arg0), medium (Lys4/Arg6) and heavy (Lys8/Arg10) SILAC labelled cells, HeLa S3 were maintained in DMEM containing 10% dialysed FBS and the corresponding Lys/Arg isotopic pair at a final concentration of 146/84 mg/l. The media were supplemented with 200 mg/l L-proline. The metabolic incorporation of the isotopes was evaluated after seven cell doublings as described^44^. For the expression of each compartment-specific Ras, labelled cells were cultured in three 15 cm dishes and transfected at 40–50% confluence with compartment-specific vectors^19^, using GeneJuice transfection reagent (Merck Millipore, Germany) according to manufacturer’s instructions. Cells were grown in SILAC medium for 20–24 hours then lysed with buffer containing 50 mM Tris-HCl pH 7.5, 100 mM NaCl, 50 mM NaF, 1% (w/v) NP-40, 0.1% (w/v) sodium deoxycholate, 1 mM EDTA, 1 mM EGTA, mammalian protease inhibitors (added according to manufacturer’s instructions), 5 mM glycerol phosphate, 2 mM sodium orthovanadate and PhosSTOP (one tablet added per 10 ml of lysis buffer) when they reached 80–90% confluence^45^. This culture configuration generated 6–7 mg of lysate from each compartment-specific Ras transfection and ensured at least 20 mg of protein for each triplex run. GFP fluorescence imaging using a Leica SP2 confocal microscope was used to confirm correct localisation and to ensure similar transfection efficiency between experiments.

### Sample preparation, LC-MS/MS and data analysis

To analyse organelle-specific Ras signalling, it was necessary to perform three triplex runs for each experiment. GFP-NRAS (G12V) was used as a common point in each triplex analysis to allow pairwise comparison between all points within an experiment. Cell lysates from three experimental conditions were mixed 1:1:1 and 150 μg and 20 mg of each protein mixture were used to characterize the proteome and phosphoproteome respectively. In-gel proteome digestion; protein digestion using the filter-aided sample preparation (FASP) protocol^46^, peptide fractionation and TiO_2_-based phosphopeptide enrichment for phosphoproteome analysis and mass spectrometry runs were carried out as described in^45^,^47^. LC-MS/MS analysis was carried out with a Waters nanoACQUITY UPCL system coupled to an LTQ Orbitrap XL (Thermo Fisher). Five μl of the phosphopeptide enriched fractions were loaded onto a 5 cm ×180 μm trap column (BEH-C18 Symmetry, Waters) and resolved on a 25 cm ×75 μm column using a 21 min linear gradient of 0–37.5% (v/v) acetonitrile in 0.1% (v/v) formic acid at a flow rate of 400 nl/min at 65 °C. For the proteomic fractions and phosphopeptide-enriched fractions from the SCX flow-through, we used 51 and 69 min linear gradients, respectively. Data acquisition was carried out as decribed in^45^,^47^. Briefly, full MS scans were acquired in the Orbitrap (*R* = 30,000; *m/z* range 300–2000) and MS^2^ scans were performed from the top 5 ions in the linear ion quadrupole ion trap after fragmentation with the collision-induced dissociation mode (30 ms at 35% energy). Dynamic exclusion of 180 s (n = 1) was applied to full MS ions previously selected for MS^2^ scans.

All raw MS files from the three biological replicates were combined and processed with the MaxQuant software suite (version 1.2.2.5)^48^,^49^. The minimum required peptide length was set to 6 amino acids and two missed cleavages were allowed. Cysteine carbamidomethylation was set as a fixed modification, whereas acetylation at the N-terminus, methionine oxidation and phosphorylation at serine, threonine and tyrosine residues were considered as variable modifications. The initial precursor and fragment ion maximum mass deviations were set to 7 ppm and 0.5 Da, respectively for the search of the IPI_HUMAN_v3.77.fasta database containing 89,709 entries. The false discovery rate (FDR) for both the peptides and proteins were set to 0.01 to ensure that the worst identified peptide/protein has a probability of 1% of being a false identification. Proteins with at least one peptide unique to the protein sequence were considered as valid identifications. SILAC ratios were considered to be significantly changing if at least a 1.5-fold change was observed.

SILAC ratios were log2 transformed, subjected to cross correlation using Excel and hierarchical clustering analysis and plotted as a heatmap using MeV (v4.8.1 software; www.tm4.org/mev). GProX was used to perform unsupervised clustering based on the fuzzy c-means algorithm^23^. The data was grouped in 6 (proteome) or 10 (phosphoproteome) clusters (fuzzification value set = 2; regulation threshold = ± 0.58; 200 iterations of the algorithm performed). The enrichment of biological features in the 6 clusters was assessed by gene ontology (GO) molecular function and GO biochemical processes annotations using DAVID Bioinformatics Database^50^ and Entrez Gene ID identifiers of shortlisted proteins and phosphosites showing ≥1.5-fold changes. Over-represented terms within the short-lists were calculated using a background list comprising all genes identified across our experiments (threshold count = 2; EASE score = 1). Terms with a *p*-value <0.05 in at least one cluster were selected, log_10_ transformed, hierarchically clustered and plotted as a heatmap. STRING v.10.0 analysis of protein-protein interactions was performed online and experimentally derived interactions used to depict the interactome^51^.

## Additional Information

**How to cite this article**: Hernandez-Valladares, M. and Prior, I. A. Comparative proteomic analysis of compartmentalised Ras signalling. *Sci. Rep.* **5**, 17307; doi: 10.1038/srep17307 (2015).

## Supplementary Material

Supplementary Figures

Supplementary Table 1

Supplementary Table 2
