# Supplementary material for: Comparative proteomic analysis of compartmentalised Ras signalling
Source: Sci Rep. 2015 Dec 1;5:17307. doi: 10.1038/srep17307 (PMC4664896; doi:10.1038/srep17307)
Supplement: Supplementary Figures [file srep17307-s1.pdf]

Comparative proteomic analysis of compartmentalised Ras signalling

Maria Hernandez-Valladares[1,2] and Ian A. Prior[1, *]

[1] Physiological Laboratory, Institute of Translational Medicine, University of Liverpool, Crown Street, Liverpool L69 3BX, United Kingdom

[2] Present address: The Proteomics Unit at the University of Bergen (PROBE), Building for Basic Biology, University of Bergen, Jonas Lies vei 91, Bergen 5009, Norway

* iprior@liv.ac.uk

Supplementary Figure 1. *Ras variant expression across the SILAC biological replicates.* GFP-Ras variant abundance normalised to the individual transfection efficiency for each condition was calculated for each of the three biological replicates. To provide an internal control for Western blotting differences, data was normalised to GFP control intensity for each gel. Mean values +/- SEM are depicted, n=3.

Supplementary Figure 2. *Proteins displaying organelle-specific responses.* (A) Proteome ratios exhibiting changes in abundance were subjected to unsupervised clustering with the Fuzzy c means algorithm using GProX. Clusters

corresponding to six different response patterns were identified. The number (n) in each cluster is indicated. (B) GO analysis indicates that proteins associated with RNA processing, gene expression and the cytoskeleton are significantly enriched in clusters 1-3, representing groups with highly variable responses between the organelle-Ras proteins, NRAS and KRAS.

Supplementary Figure 3. *GProX cluster 3 and cluster 5 phosphosite responses.* Members of cluster 3 and 5, enriched in cytoskeletal and DNA organising proteins highlighted in a representative KRAS versus ER/Golgi-Ras and Golgi-Ras versus ER/Golgi-Ras scatter graphs.

Supplementary Figure 4. *Kinase responses within the proteomic datasets.* All kinase protein (A) and kinase phosphosite (B) responses versus GFP control within the datasets.

Supplementary Table 1. *Compartmentalised Ras MaxQuant data sets.* Phosphosite and proteome ratios for the combined MaxQuant analysis of all 3 SILAC biological replicates. Ratios for Organelle Ras/NRAS and Organelle Ras/GFP control as indicated.

Supplementary Table 2. *Compartmentalised Ras responsive proteins and phosphosites.* All phosphosites and proteome proteins exhibiting ≥1.5-fold

changes versus GFP control in at least one of the 6 experimental conditions (ER/Golgi, endo, KRAS, NRAS, Golgi, mito).

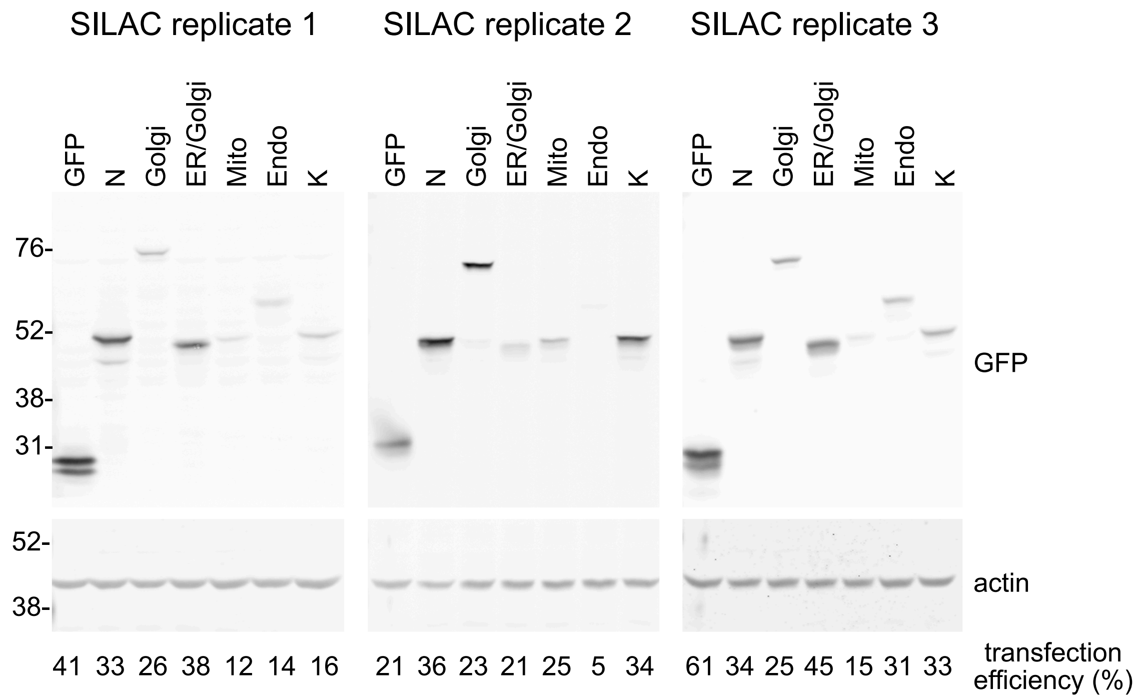

SILAC replicate 1
SILAC replicate 2
SILAC replicate 3
GFP
N
Golgi
ER/Golgi
Mito
Endo
K
76-
52-
38-
31-
52-
38-
GFP
actin
41 33 26 38 12 14 16
21 36 23 21 25 5 34
61 34 25 45 15 31 33
transfection efficiency (%)

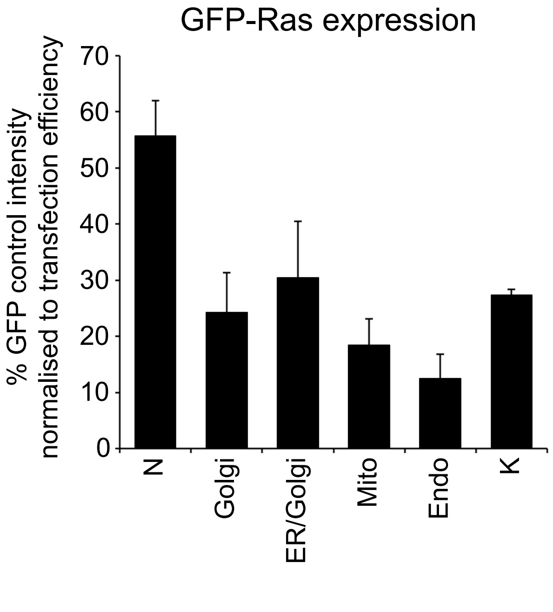

GFP-Ras expression
% GFP control intensity normalised to transfection efficiency
0
10
20
30
40
50
60
70
N
Golgi
ER/Golgi
Mito
Endo
K

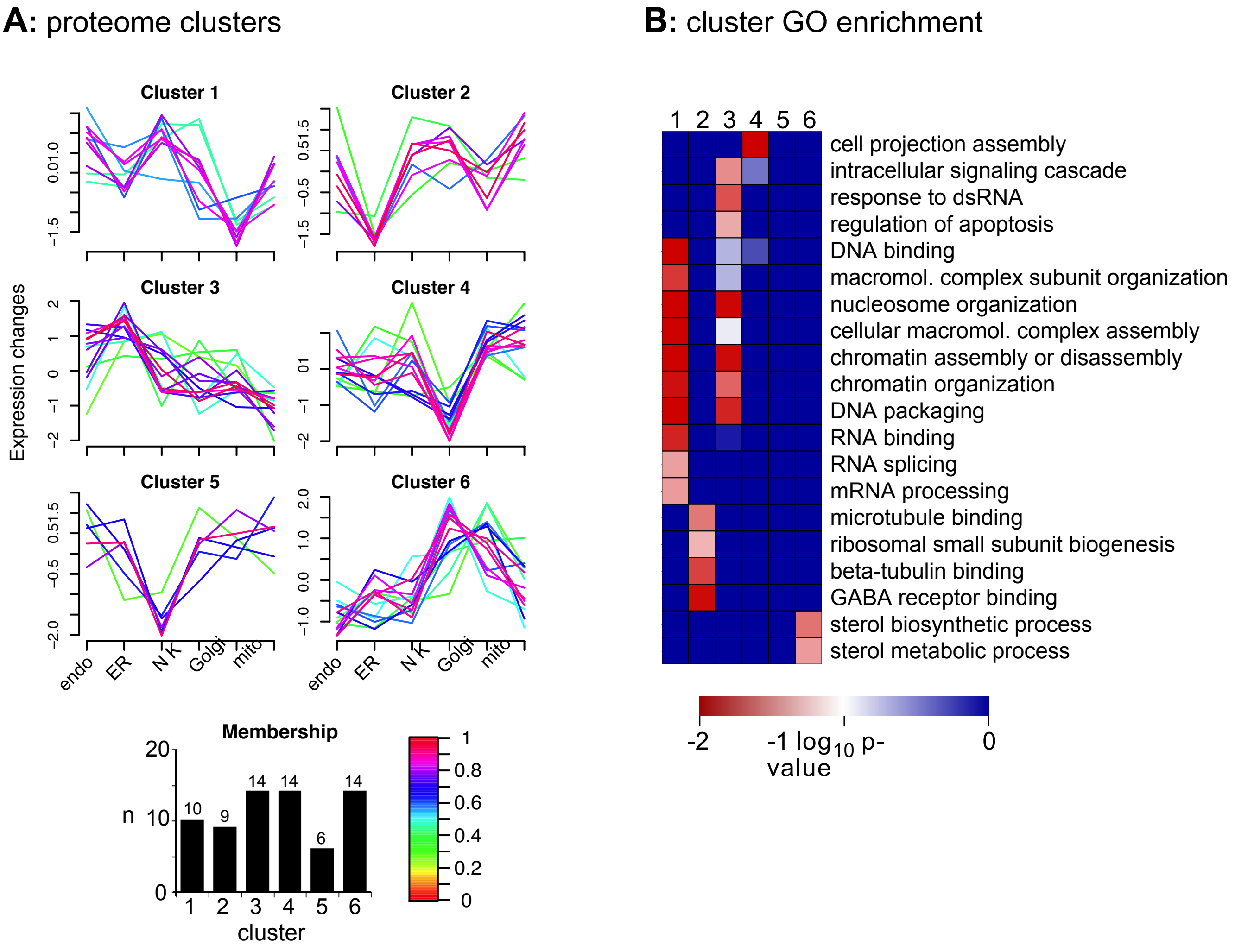
A: proteome clusters
Cluster 1
Cluster 2
Cluster 3
Cluster 4
Cluster 5
Cluster 6
Expression changes
endo
ER
N K
Golgi
mito
Membership
n
cluster
B: cluster GO enrichment
1 2 3 4 5 6
cell projection assembly
intracellular signaling cascade
response to dsRNA
regulation of apoptosis
DNA binding
macromol. complex subunit organization
nucleosome organization
cellular macromol. complex assembly
chromatin assembly or disassembly
chromatin organization
DNA packaging
RNA binding
RNA splicing
mRNA processing
microtubule binding
ribosomal small subunit biogenesis
beta-tubulin binding
GABA receptor binding
sterol biosynthetic process
sterol metabolic process
-2
-1 log10 p-value
0

cluster 3 **cluster 5**

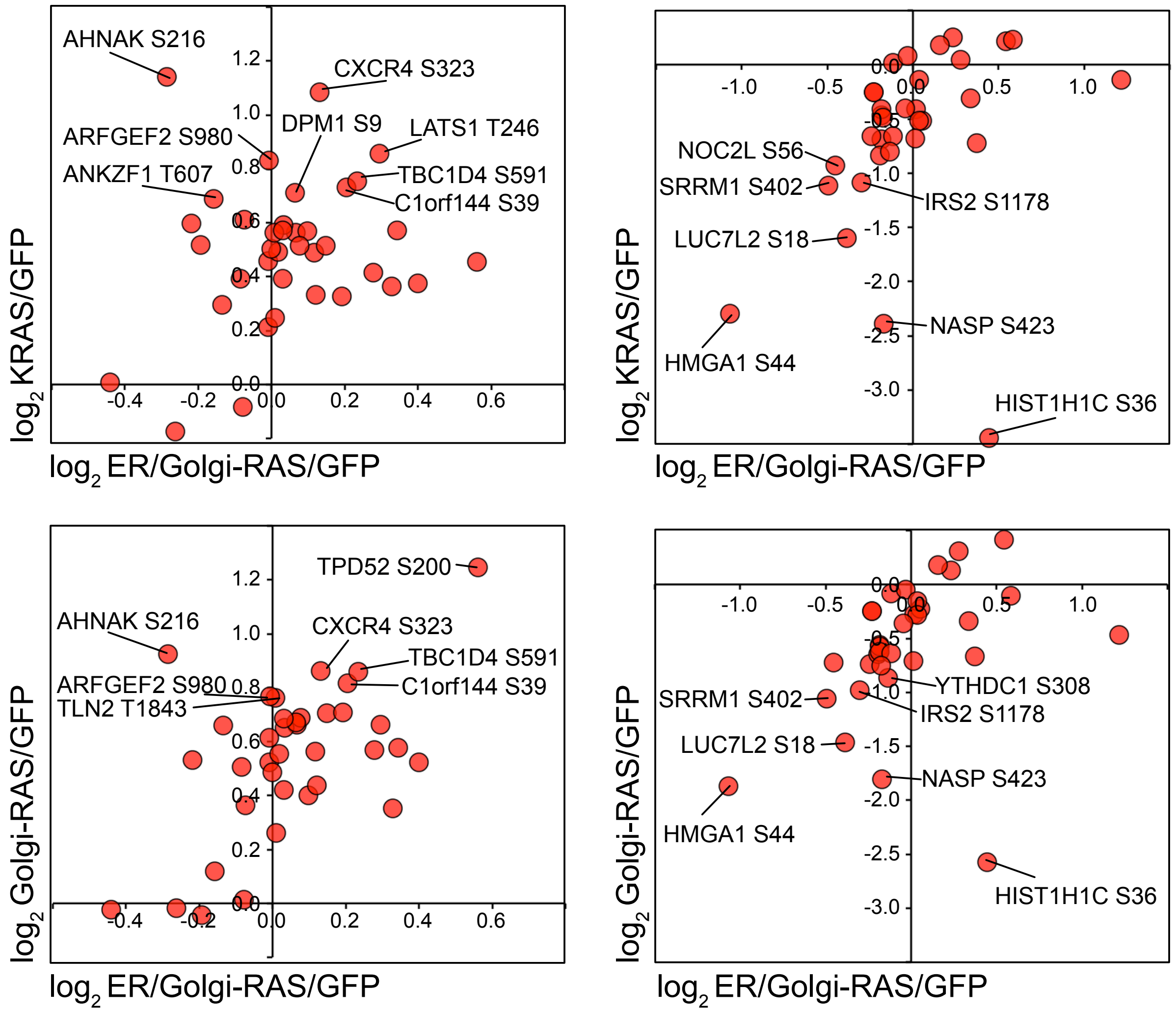

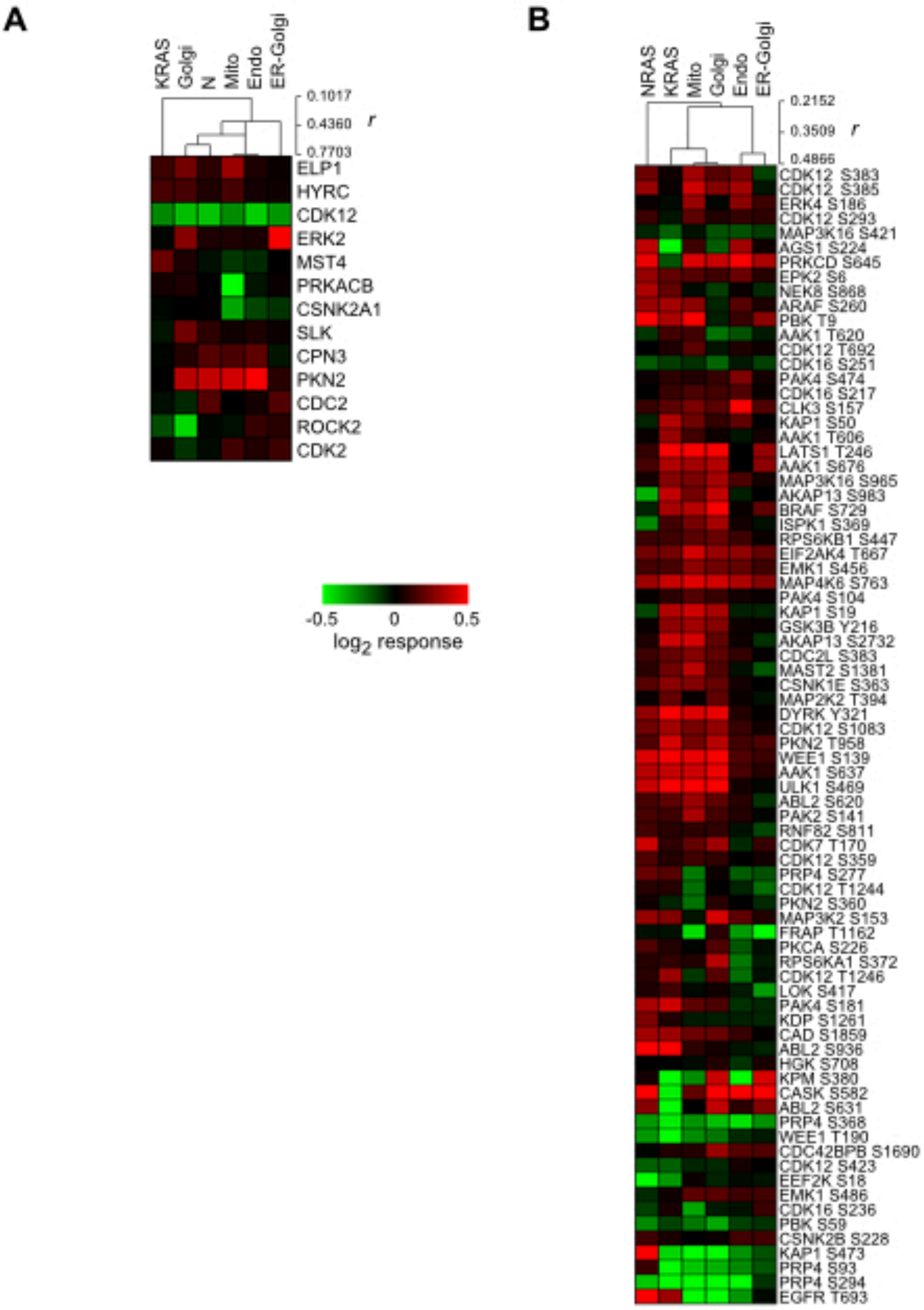
A
KRAS
Golgi
N
Mito
Endo
ER-Golgi
0.1017
0.4360
r
0.7703
ELP1
HYRC
CDK12
ERK2
MST4
PRKACB
CSNK2A1
SLK
CPN3
PKN2
CDC2
ROCK2
CDK2
-0.5
0
0.5
log2 response
B
NRAS
KRAS
Mito
Golgi
Endo
ER-Golgi
0.2152
0.3509
r
0.4866
CDK12 S383
CDK12 S385
ERK4 S186
CDK12 S293
MAP3K16 S421
AGS1 S224
PRKCD S645
EPK2 S6
NEK8 S868
ARAF S260
PBK T9
AAK1 T620
CDK12 T692
CDK16 S251
PAK4 S474
CDK16 S217
CLK3 S157
KAP1 S50
AAK1 T606
LATS1 T246
AAK1 S676
MAP3K16 S965
AKAP13 S983
BRAF S729
ISPK1 S369
RPS6KB1 S447
EIF2AK4 T667
EMK1 S456
MAP4K6 S763
PAK4 S104
KAP1 S19
GSK3B Y216
AKAP13 S2732
CDC2L S383
MAST2 S1381
CSNK1E S363
MAP2K2 T394
DYRK Y321
CDK12 S1083
PKN2 T958
WEE1 S139
AAK1 S637
ULK1 S469
ABL2 S620
PAK2 S141
RNF82 S811
CDK7 T170
CDK12 S359
PRP4 S277
CDK12 T1244
PKN2 S360
MAP3K2 S153
FRAP T1162
PKCA S226
RPS6KA1 S372
CDK12 T1246
LOK S417
PAK4 S181
KDP S1261
CAD S1859
ABL2 S936
HGK S708
KPM S380
CASK S582
ABL2 S631
PRP4 S368
WEE1 T190
CDC42BPB S1690
CDK12 S423
EEF2K S18
EMK1 S486
CDK16 S236
PBK S59
CSNK2B S228
KAP1 S473
PRP4 S93
PRP4 S294
EGFR T693
